# A phase II study of concurrent chemoradiotherapy combined with a weekly paclitaxel and 5-fluorouracil regimen to treat patients with advanced oesophageal carcinoma

**DOI:** 10.1186/s13014-017-0785-0

**Published:** 2017-03-07

**Authors:** Yi Xia, Yun-hai Li, Yun Chen, Jun-hua Zhang, Qi Liu, Jia-ying Deng, Ta-shan Ai, Han-ting Zhu, Jian-hong Fan, Harun Badakhshi, Kuai-le Zhao

**Affiliations:** 1 Department of Radiation Oncology, Fudan University, Shanghai Cancer Center, 270 Dong’An Road, Shanghai, 200032 China; 2 Department of Oncology, Shanghai Medical College, Fudan University, Shanghai, China; 3 Department of Radiation Oncology, Fudan University, Shanghai Cancer Center Minhang Branch, Shanghai, China; 4 Department of Gynaecology, Renhe hospital, Baoshan district Shanghai, China; 5 Department of Radiation Oncology, Charité School of Medicine and Centre for Cancer Medicine, Berlin, Germany

**Keywords:** Oesophageal carcinoma, Paclitaxel, 5-Fluorouracil, Chemoradiotherapy

## Abstract

**Background:**

A phase II study was performed to investigate the safety and efficacy of weekly doses of combined paclitaxel and 5-fluorouracil (5-FU) with concurrent radiation therapy, followed by 2 cycles of consolidation chemotherapy to treat patients with advanced oesophageal carcinoma.

**Methods:**

The eligibility criteria included local, advanced, newly diagnosed and postoperative local regional lymph node metastasis; an Eastern Cooperative Oncology Group (ECOG) score of ≤ 2; and adequate organ function. Patients received chemoradiotherapy consisting of radiotherapy (50.4 Gy/28 Fx or 61.2 Gy/34 Fx) and concurrent paclitaxel (50 mg/m^2^) and 5-FU (300 mg/m^2^) for 96 h on days 1, 8, 15, 22, and 29. The two-cycle consolidation chemotherapy protocol included paclitaxel (175 mg/m^2^) plus continuously infused 5-FU (1800 mg/m^2^) for 72 h administered on days 57 and 85, after concurrent chemoradiotherapy.

**Results:**

Between February 2012 and August 2013, 53 patients with oesophageal carcinoma were enrolled in the study. Among these patients, 33 (62.2%) were newly diagnosed and 20 (37.7%) had postoperative local regional lymph node metastasis. The median overall survival (OS) time was 17.9 months (95% CIs = 11.9-23.9), and the median progression-free survival (PFS) time was 12.4 months (95% CIs = 8.6-16.1). Approximately 84.9% (45/53) and 50.9% (27/53) of the patients completed ≥ 5 cycles and all 7 cycles of chemotherapy, respectively. Approximately 86.7% (46/53) of patients completed radiation therapy. The 1-, 2-, and 3-year OS rates were 66.0%, 37.7%, and 35.8%, respectively. The 1-, 2-, and 3-year local control rates were 76.9%, 66.4%, and 66.4%, respectively. Seventeen patients (32%) experienced grade 3 or higher toxicity. Grade 3 to 5 toxicity during chemoradiotherapy included neutropaenia (7.5%), thrombocytopaenia (1.8%), fatigue (7.5%), anaemia (1.8%), dermatitis radiation (1.8%), pneumonitis (5.6%), oesophagitis (9.4%) and vomiting (3.7%).

**Conclusions:**

The combination of weekly doses of paclitaxel and 5-FU was well tolerated and produced comparable results among patients with locally advanced oesophageal cancer. A randomised phase III trial (NCT01591135) comparing paclitaxel plus 5-FU with cisplatin plus 5-FU is on-going at our hospital.

## Background

Oesophageal cancer is the fifth most common type of cancer and fourth most common cause of cancer-related death in China. This cancer is highly malignant because of high rates of local recurrence and metastasis. Definitive concurrent chemoradiotherapy is the standard treatment for non-surgery patients with oesophageal cancer, and the use of cisplatin with 5-fluorouracil (PF) is the most common chemotherapy regimen [1]. However, 5-year survival rates are poor (26%) following definitive chemoradiation therapy [2]. Thus, more sensitive, less toxic chemotherapy regimens are urgently needed.

Paclitaxel is a promising agent with regard to oesophageal cancer, with response rates of approximately 32% via single-drug treatment in locally advanced and metastatic patients [3]. Paclitaxel is a mitotic inhibitor that blocks cells during the G2M-phase of the cell cycle (i.e., the most radiosensitive phase), with a sensitising enhancement ratio of 1.48 [4]. The combination of paclitaxel and cisplatin (TP) is the most commonly used regimen, and the response rates among advanced and metastatic patients with oesophageal cancer are approximately 46%–59% [5–7], with a median survival of 6.9 to 13 months [5, 6, 8]. Radiotherapy concurrent with a monthly schedule of TP resulted in 3-year overall survival (OS) and local failure rates of 41% and 27.6% but a relatively higher haematological toxicity, resulting in neutropaenia grades 3 and 4 in 30.3% and 31.6% of cases, respectively [9]. Increased toxicity with no improvement in OS compared with the PF regimen was associated with the TP regimen for advanced oesophageal cancer [10].

The investigation of a new paclitaxel-based regimen and administration route is important for improving survival and reducing side effects. Effective and well-tolerated chemotherapy can prolong OS and improve quality of life. A pilot study of concurrent paclitaxel and 5-FU plus radiotherapy was developed for patients with localised oesophageal cancer at the University of Texas MD Anderson Cancer Center [11]. In that study, the continuous infusion of 5-FU and paclitaxel combined with radiotherapy 5 times was well tolerated; toxicities included mild vomiting, nausea, oesophagitis, and abdominal pain. The combination of paclitaxel and 5-FU was tolerated and effective. Based on these data, we began a phase II feasibility study to evaluate the failure-free local control rate, OS, and toxicities in patients with advanced oesophageal cancer treated with weekly paclitaxel and 5-FU.

## Methods

### Patient selection

The eligibility criteria for the study were as follows: (1) age ≤ 75 years; (2) Eastern Cooperative Oncology Group (ECOG) score 0–2; (3) cytologically or histologically confirmed oesophageal cancer; and (4) locally advanced, newly diagnosed patients with T2-4NxM0-1a or TxNxM1b (supraclavicular lymph node metastases for middle and lower thoracic oesophageal cancer or mediastinal lymph node metastases for cervical oesophageal cancer without organ metastases according to the 6th edition of UICC) or postoperative patients with local regional lymph node metastasis who did not receive chemotherapy or radiotherapy. Lymph nodes found via imaging increased gradually over the follow-up period, and the diameters of these nodes were more than 1 cm. This sign was considered to indicate postoperative lymph node relapse. Not all patients required a PET scan.

Patients were required to meet the following laboratory criteria: adequate bone marrow function (neutrophil count > 2.0*10^9^/L, white blood cell count > 4.0*10^9^/L, and platelet count > 100*10^9^/L), normal renal function, and normal liver function. Patients with tracheoesophageal fistula, organ metastasis, or complete obstruction were not eligible for this study. All patients underwent a complete examination and nutritional assessments. The disease evaluation included an upper endoscopy, chest and abdominal computed tomography (CT), ultrasonography, and barium oesophagram examination to eliminate distant organ metastasis.

### Ethical Considerations

Written informed consent was obtained from all patients before pre-study assessments, and the Ethics Committee of the Cancer Hospital Affiliated to Fudan University approved the study protocol.

### Treatment schedule

**Fig. 1:**
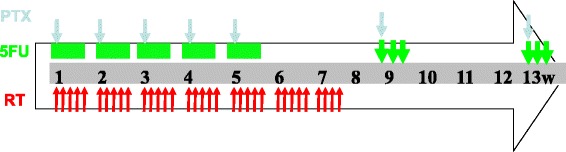
Treatment schedule

The chemotherapy regimen consisted of a combination of paclitaxel and 5-FU. The treatment modality is illustrated in Fig. 1. Concurrent chemotherapy was administered from the first day of radiotherapy and included five cycles of paclitaxel (50 mg/m^2^) over a 3-h infusion and continuously infused 5-FU (300 mg/m^2^) for 96 h on days 1, 8, 15, 22 and 29. Thirty minutes before treatment with paclitaxel, patients were pre-medicated with 10 mg of dexamethasone and 300 mg of cimetidine intravenously and 25 mg of promethazine intramuscularly. The two-cycle consolidation chemotherapy protocol was paclitaxel (175 mg/m^2^) plus continuously infused 5-FU (1,800 mg/m^2^) for 72 h on days 57 and 85. The pre-treatment before paclitaxel treatment was as follows: 25-mg promethazine intramuscular injection at 0.5 h, 27 tablets of dexamethasone administered orally (0.75 mg/tablet) at 12 and 6 h before treatment, and 300 mg of cimetidine administered intravenously at 0.5 h before paclitaxel treatment. If a grade of ≥ 3 haematological toxicity occurred and persisted, then chemotherapy was suspended until recovery, and the regimen dose was sequentially reduced by 25%.

Radiation therapy was administered using 3-dimensional conformal radiation therapy (3DCRT) or intensity-modulated radiation therapy (IMRT). Planning was performed using a Megavoltage simulator with a photon energy of 6 MV. For previously untreated diagnosed patients and recurrent patients, the gross tumour volume (GTV) was defined as the volume of the primary tumour observed on the oesophageal barium exam, upper digestive endoscopy and CT. Metastatic lymph nodes were defined as lymph nodes ≥ 1 cm in the shortest axis and ≥ 5 mm in the tracheoesophageal groove. The clinical target volume (CTV) was defined by adding 3-cm margins of proximally and distally uninvolved oesophagus without including the lateral margins and lymph nodes. The planning target volume (PTV) was calculated by adding 1-cm margins around the CTV, but the margins were reduced when the PTV recovered the spinal cord. All patients were treated with a total dose of either 61.2 Gy in 34 fractions or 50.4 Gy in 28 fractions. The lower dose was used when patients had abdominal lymph nodes metastasis or tumour in excess of the limitations of normal organs such as the lungs, spiral cord, or intestines.

The plan optimisation was as follows: (1) 99% of the PTV was covered by 95% of the prescribed dose; (2) 95% of the PTV volume was covered by the prescribed dose; (3) the maximum dose did not exceed 110% of the prescribed dose in a continuous volume of < 1 cm^3^ in the PTV; and (4) the maximum dose of the PTV did not exceed 110% of the prescribed dose in a continuous volume of < 1 cm^3^. The normal tissue constraints of the critical organs were as follows: a maximum spiral cord point dose of ≤ 45 Gy; a percentage of total lung volume receiving ≥ 20 Gy (lung V20) of ≤ 30% and a mean lung of ≤ 16 Gy, mean heart dose of ≤ 30 Gy, and maximum intestine dose of ≤ 50 Gy.

### Assessment of primary tumour response

The response of the primary tumour was determined 3 months after the last cycle of chemotherapy was completed. Patients were scheduled to have CT scans and barium oesophagrams. If no obvious disease progression or symptoms were present, then the disease was considered to be controlled. If the disease progressed, then patients were scheduled to have endoscopy/biopsy, needle biopsy, or endobroncheal ultrasonography (EBUS). However, some of the patients who showed disease progression could not be given a clear pathological diagnosis because of the location of the tumour; these patients were diagnosed via imaging.

### Follow up

Over the first year after treatment completion, the patients returned to the outpatient clinic every 3 months for a history and clinical examination. During the second year after treatment, the follow-up assessments occurred every 6 months and then every year until year 5. When applicable, disease recurrence, late toxic effects, and death were documented. Adverse reactions to chemoradiotherapy were evaluated according to the National Cancer Institute Common Toxicity Criteria version 3.0 (NCI-CTC 3.0).

### Statistical methods

The primary end point of this study was the failure-free local control rate. The secondary end points included assessments of safety and compliance, OS, and progression-free survival (PFS). Local failure was defined as the recurrence or persistence within the radiation therapy PTV. Recurrence outside the treatment volume was defined as distant. OS was calculated from the first day of chemoradiation therapy to the time of the last follow-up assessment or death. PFS was defined as the time from chemoradiotherapy day 1 to progression, death, or last follow-up assessment. We hypothesised that the addition of paclitaxel and 5-FU would increase the failure-free local control rate. A sample size of 53 was required to detect an increase in the local control rate from 48% to 65% with a power of 80% and a standard error of 0.05. The data were analysed according to the intention-to-treat model. Kaplan-Meier curves were fit to estimate the OS and failure-free local control rates. Median survival estimates were calculated. Statistical analyses were performed using SPSS 20.0.

## Results

### Patient characteristics

**Fig. 2:**
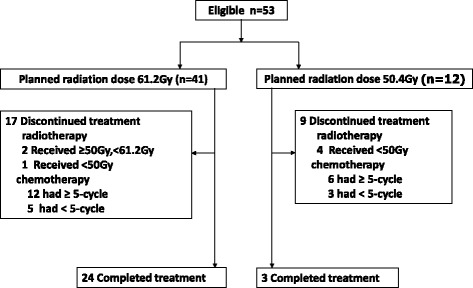
Consort diagram of patient progress throughout the study

Between February 2012 and August 2013, 53 patients were enrolled in our study. Figure 2 shows the progression across the study phases. The baseline characteristics of the 53 patients are shown in Table 1. The median patient age was 59 years (range = 42–73). Six patients (11.3%) had other malignant tumours, and 46 patients (87.8%) were men. The ECOG performance statuses of 48 and 5 patients were 0–1 and 2, respectively. The most common tumour histology was squamous (94.3%). Of all patients, 33 (62.2%) were newly diagnosed, and 20 (37.7%) had postoperative regional lymph node metastasis and had not received prior chemotherapy or radiotherapy. Of the 33 newly diagnosed patients, 6 (18.1%) were stage II, 10 (30.3%) were stage III, and 17 (51.5%) were stage IV. Two had tumours in their cervical regions, 7 had tumours in their upper thoracic regions, 9 had tumours in their middle thoracic regions, 9 had tumours in their lower thoracic regions, and 6 had tumours in multiple synchronous primary sites. The median interval between surgery and relapse concerning the 20 patients with postoperative lymph node relapse was 15.2 months. Twenty-one and 32 patients were treated using IMRT and 3D-CRT, respectively. The medians for GTV and PTV were 55.2 cm^2^ and 410.7 cm^3^, respectively.

**Table 1:** Patient characteristics (*N* = 53)

Characteristics	No.of patients (%)
*Age (years)*	
Median	59
Range	42-73
*Gender*	
Male	46 (87.8)
Female	7 (13.2)
*Histology*	
Squamous	50 (94.3)
Adenocarcinoma	3 (5.7)
*Tumor location of newly diagnosed*	33 (62.2)
Cervical	2 (3.8)
Upper thoracic	7 (13.2)
Middle thoracic	9 (17.0)
Lower thoracic	9 (17.0)
Synchronous multiple primary	6 (11.3)
*ECOG* ^*a*^ *score*	
0-1	48 (90.6)
2	5 (9.4)
*Stage (UICC* ^*b*^ *6* ^*th*^ *edition)*	
Newly diagnosed	33 (62.2)
T4NxM0	9 (17.0)
T2-3NxM0-1	17 (32.0)
T4NxM1	7 (13.2)
Postoperative LNM^c^	20 (37.7)
*Planned dose of radiation*	
61.2 Gy	41 (77.4)
50.4 Gy	12 (22.6)

^a^Eastern Cooperative Oncology Group; ^b^Union for International Cancer Control; ^C^lymph node metastasis

### Local control and OS rates

**Fig. 3:**
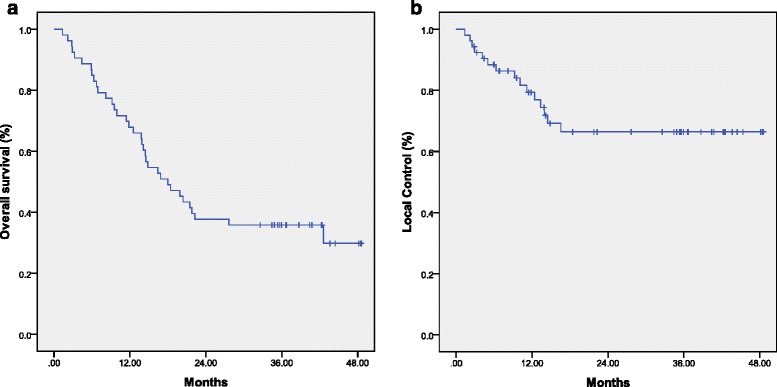
OS (**a**) and local control rate (**b**) curves for all patients

The median follow-up time was 38.4 months. At the time of our analyses, 20 patients were living, and 15 patients showed no evidence of disease progression. The 1-, 2-, and 3-year OS rates were 66.0%, 37.7%, and 35.8%, respectively. The 1-, 2-, 3-year local control rates were 76.9%, 66.4%, and 66.4%, respectively. The median OS time was 17.9 months (95% CIs = 11.9-23.9), and the median PFS time was 12.4 months (95% CIs = 8.6-16.1). The Kaplan-Meier curves for the failure-free local control rates and OS times are shown in Fig. 3. The 3-year OS rate was 27.3% for the newly diagnosed group and 50.0% for the postoperative recurrent group, with median survival times of 16.4 and 21.7 months, respectively (*P* = 0.235). The mean OS was 21.4 months for the radiation dose of 61.2 Gy and 12.5 months for the dose of 50.4 Gy (*P* = 0.011). A total of 38 patients (71.7%) showed treatment failure. Ten patients showed only locoregional failure, 22 patients showed only distant metastasis, 4 patients showed concurrent locoregional/distant failure, and 2 patients failed therapy due to radiation toxicity. The treatment failure patterns of the 38 patients are presented in Table 2. At the time of the analysis, 26 patients had distant metastases. The sites of the metastases included the lungs (14 patients, 26.4%), bones (10 patients, 18.8%), liver (6 patients, 11.3%), skin (2 patients, 3.7%), adrenal glands (2 patients, 3.7%), and distant lymph nodes (12 patients, 22.6%). Of the 14 cases of locoregional failure, 10 patients showed local/regional recurrence, and 4 showed persistent local/regional disease (3 patients did not complete radiotherapy).

**Table 2:** Patterns of failure

	No.	Percent
Any failure	38	71.7
Locoregional failure only	10	18.9
Distant failure only	22	41.5
Locoregional plus distant	4	7.5
Toxicity	2	3.7

### Feasibility and toxicity

Of all patients, 41 had a planned radiation dose of 61.2 Gy, and 38 (92.6%) completed radiotherapy treatment. Of the 12 patients with a planned radiation dose of 50.4 Gy, 8 (66.6%) finished the complete radiotherapy regimen. A total of 46 patients (86.7%) completed treatment based on our definition for completing the radiotherapy regimen. Seven (13.2%) patients did not finish radiotherapy because of grade II toxicity to the skin (1 patient), grade III cough and haemoptysis (1), fever lasting 10 days (1), grade III vomiting (1), obstruction of the oesophagus (2), or death from gastrointestinal bleeding (1). A ≥ 5-cycle dose of concurrent chemotherapy combined with paclitaxel and 5-FU during radiotherapy was administered to 45 patients (84.9%), and a full dose of 7 cycles of chemotherapy was administered to 27 patients (50.9%). Eight patients (15.1%) did not finish the 5-cycle dose of concurrent chemotherapy because of obstruction of the oesophagus (2), disease progression (1), adverse reactions (4), or death from gastrointestinal bleeding (1). The gastrointestinal bleeding was considered to be caused by a tumour.

The toxicity and tolerability of the therapy were evaluated in all patients. The toxicities of chemoradiotherapy (graded according to NCI-CTC 3.0) are outlined in Table 3. Seventeen patients (32%) experienced grade 3 or higher sever events in this study. The most frequent acute adverse events for grades 3 and 4 were neutropaenia, oesophagitis, and fatigue. Grades 3 and 4 neutropaenia were observed in 3 (5.6%) patients and 1 (1.8%) patient, respectively. Grades 3 and 4 thrombocytopaenia, anaemia, fatigue, and oesophagitis occurred in 1/53 (1.8%), 1/53 (1.8%), 4/53 (7.5%), and 5/53 (9.4%) cases, respectively. Eight (15.0%) of the 53 patients experienced fever in addition to the patients who experienced radiation pneumonitis. Three of the 8 patients with fever experienced febrile neutropaenia. The other types of adverse reactions related to treatment were mild and included hypoalbuminaemia, anorexia, hoarseness, and pericardial effusion. No allergic reaction was recorded in any of the patients. The grade ≥ 3 pneumonitis and dermatitis radiation rates were 5.6% (3/53) and 1.8% (1/53), respectively. The 2 treatment-related deaths among the 53 patients were because of radiation pneumonitis. Three patients showed pericardial effusion without symptoms. For 2 of these patients, effusion occurred within 3 months after completing radiotherapy. For the remaining patient, effusion occurred within 6 months of completing radiotherapy.

**Table 3:** Treatment toxicity^a^

Grade	1	2	3	4	5
N = 53	No. (%)	No. (%)	No. (%)	No. (%)	No. (%)
Neutropaenia	21 (39.6)	8 (15.0)	3 (5.6)	1 (1.8)	0
Thrombocytopaenia	9 (16.9)	4 (7.5)	1 (1.8)	0	0
Anaemia	21 (39.6)	8 (15.0)	0	1 (1.8)	0
Vomiting	9 (16.9)	3 (5.6)	1 (1.8)	0	0
Fever^b^	7 (13.2)	1 (1.8)	0	0	0
Fatigue	14 (26.4)	3 (5.6)	3 (5.6)	1 (1.8)	0
Alopecia	9 (16.9)	6 (11.3)	0	0	0
Hypoalbuminaemia	7 (13.2)	2 (3.7)	0	0	0
ALT^c^	9 (16.9)	3 (5.6)	1 (1.8)	0	0
Anorexia	16 (30.1)	11 (20.7)	1 (1.8)	0	0
Dermatitis radiation	10 (18.8)	5 (9.4)	1 (1.8)	0	0
Oesophagitis	25 (47.1)	16 (30.1)	5 (9.4)	0	0
Hypocalcaemia	6 (11.3)	2 (3.7)	0	0	0
Hypomagnesaemia	1 (1.8)	0	0	0	0
Hyponatraemia	2 (3.7)	0	2 (3.7)	0	0
Hypophosphataemia	3 (5.6)	0	0	0	0
Constipation	2 (3.7)	2 (3.7)	0	0	0
Diarrhea	3 (5.6)	0	0	0	0
Peripheral sensory neuropathy	13 (24.5)	4 (7.5)	0	0	0
Pneumonitis	5 (9.4)	0	1 (1.8)	0	2 (3.7)
Hypokalaemia	3 (5.6)	0	1 (1.8)	0	0
Hoarseness	5 (9.4)	0	0	0	0
Ventricular arrhythmia	1 (1.8)	0	0	0	0
Creatinine increased	1 (1.8)	0	0	0	0
Pericardial effusion	2 (3.7)	0	0	0	0

^a^Toxicity was grade according to the National Cancer Institute Common Toxicity Criteria, version 3.0; ^b^Of the 8 patients, 3 patients experienced febrile neutropenia; ^C^Alanine aminotransferase increased

## Discussion

In our phase II study, the regimen of concurrent weekly paclitaxel, 5-FU, and radiotherapy showed promise for treating advanced oesophageal cancer in terms of the local control rate, OS, and low rate of side effects. Seventeen patients (32%) experienced grade 3 or higher sever events in this study. The common adverse reactions were bone marrow suppression, oesophagitis, fatigue, and pneumonitis. The 1- and 3-year local control rates were 76.9% and 66.4%, respectively. The median OS time was 17.9 months. The 1- and 3-year OS rates were 66.0% and 35.8%, respectively, although 69.8% (37/53) of the patients in this study were stage IV and showed recurrence.

Based on the 3-year follow-up results of our study and compared with other studies of PF concurrent radiotherapy, the paclitaxel and 5-FU regimen might have produced less toxicity and increased local control. In the RTOG 8501 study [2], local disease failure was the greatest cause of treatment failure and comprised 54% of the PF combined regimen therapy group. The median survival duration was 14.1 months with a 5-year survival rate of 26% in the chemoradiotherapy treatment group. In addition, grades 3 and 4 adverse reactions occurred in 42% and 4% of the patients, respectively. In the RTOG9405 study [12], patients were treated with radiotherapy doses of 64.8 and 50.4 Gy with DDP plus 5-FU, and the median survival times were 13.0 and 18.1 months, respectively. The incidence of local failure was 56% in the high dose group and 52% in the standard dose group. The RTOG9405 study was composed of 7.7% T4 patients and 21.5% N1 patients, and the incidences of grade 3 to 5 acute toxicity in the high-dose/low-dose groups were 76% and 71%, respectively. In our study, however, all patients had non-resectable or recurrent oesophageal cancer, including 17/33 (51.5%) previously untreated patients diagnosed with stage IV disease. Ohtsu et al. reported the use of chemoradiotherapy for oesophageal cancer with T4, M1 lymph node involvement, or both. In this study, the pattern of first failure was 57.4% (31/54) locoregional and 38.8% (21/54) distant, and the treatment resulted in a median survival duration of 9 months with a 3-year survival rate of 23% [13]. In our study, the median OS was 17.9 months, and the 1- and 3-year failure-free local control rates were 76.9% and 66.4%, respectively, which represented an improvement compared with the results reported in the PF regimen study.

Paclitaxel and cisplatin (TP) regimens are the most commonly reported paclitaxel-based regimens. The grade ≥ 3 haematological toxicity rates of TP are 30.6-73.7% [5, 9, 14]. In our study, the grade ≥ 3 haematological toxicity rate was 11.3%, which was lower than the results reported in a TP-based group study. Adelstein et al. reported the use of TP in a phase II study [10]. The median survival was 15 months for patients with stage T3, N1 or M1 (nodal) oesophageal cancer, which was lower than that observed in our study. Neutropaenia (95%) and nausea (95%) were the most frequent grade III/IV toxicities, and 16 patients (40%) experienced neutropaenic fever, which must be addressed. This toxicity is significant and was higher than that in our study. Tu et al. reported a retrospective study of combined-modality therapy consisting of the TP regimen with a concurrent IMRT of 60 Gy [14]. The median OS was 18.0 months, and grade 3 haematological and radiodermatitis toxicities were observed in 11 (30.6%) and 8 (22.2%) patients, respectively. That study was composed of 66.7% stage II-III patients. By contrast, all patients in the present study had non-resectable or recurrent oesophageal cancer, and only 16/53 (30.1%) patients were stage II-III. Tang et al. reported a phase II study of concurrent chemoradiotherapy with a 3-week schedule of TP for oesophageal cancer [9]. The median OS was 28.5 months; however, neutropaenia grade 3-4 occurred in 61.9% of the patients, with relatively higher haematological toxicity. In the RTOG 0113 study, one group was treated with TP and a radiotherapy dose of 50.4 Gy, and grades 3 and 4 adverse reactions occurred in 43% and 40% of the patients, respectively [15]. However, another group was treated with paclitaxel and 5-FU and exhibited lower treatment-related mortality and less grade 4 toxicity than a group treated with TP. These results suggest that compared with the cisplatin and 5-FU regimen containing paclitaxel, the adverse reactions in our study associated with paclitaxel and 5-FU were relatively low. The TP regimen might have higher haematological toxicity, similar to the results of a previous phase II study of concurrent chemoradiotherapy with TP for patients with inoperable oesophageal cancer [9].

The Chemotherapy for Oesophageal Cancer Followed by Surgery Study (CROSS) showed that neoadjuvant chemoradiation combined with carboplatin and paclitaxel concurrent with radiation significantly prolonged OS compared with surgery alone. Neoadjuvant chemoradiotherapy combined with a paclitaxel and carboplatin regimen did not increase surgery-related mortality. Thus, preoperative chemotherapy combined with carboplatin and paclitaxel followed by surgery should be considered as a standard of care among patients with resectable oesophageal cancer. A randomised phase III trial (NCT02459457) with a larger sample size is currently examining the efficacy of paclitaxel plus 5-FU compared with paclitaxel plus carboplatin concurrent with radiotherapy among patients with local advanced oesophageal cancer at our hospital.

While undergoing chemoradiotherapy in our study, 8 patients experienced fever, and 3 of these patients experienced febrile neutropaenia. The rate of fever was higher than expected and higher than the rates reported in other studies [9, 16, 17]. We do not have a reasonable explanation for this discrepancy, and the patient with a fever caused by pneumonitis without additional symptoms was treated with a similar approach as the fever patients and was not evaluated via chest CT. The grade 5 radiation pneumonitis rate was 3.7% (2/53), which was higher than that in other studies [9, 17, 18]. The 2 patients with grade 5 radiation pneumonitis completed concurrent chemotherapy, and both died within 3 months of undergoing the radiotherapy regimen; one patient with T4 received 61.2 Gy of radiation therapy, and the another with M1 received 50.4 Gy of radiotherapy. The lung doses associated with the two deaths were within the normal constraints. The lung V20 and mean lung dose of the two deaths were 19.1% and 23.4%, 11.6 Gy and 15.2 Gy, respectively. We do not have a reasonable explanation for this finding, which might be attributable to the small number of patients or the late stage of cancer. We believe that the lung dose was only one of the factors that affected the occurrence of radiation pneumonia. One study reported that 7% of patients with late-stage cancer succumbed to treatment-related death after chemoradiotherapy [13]. Similar to other reports [19, 20], 84.9% (45/53) of the patients in our study completed a concurrent chemoradiotherapy regimen. However, 18 patients did not complete the consolidation chemotherapy because they were unwilling (5) or experienced disease progression (5) or an adverse reaction (8). The completion rate of the full dose of 7 cycles of chemotherapy was lower than previous reports [19, 20]. Compared with patients in the later stages, their tolerance was poor.

The limitations of our study are as follows. 1) The homogeneity of the patients in the group was low; patients in phases II-III and IV were included, and postoperative recurrence was present. 2) The radiation dose was not uniform. Although the standard radiotherapy dose is 50.4 Gy in Europe and the United States, the radiotherapy dose for oesophageal cancer in China is often 60–70 Gy. Normal tissue could not tolerate the use of 50.4 Gy in our study. Wang et al. reported that patients who received a radiation dose of ≥ 50 Gy showed a better outcome than those who received < 50 Gy [20]. Regarding the limitations of our study, a randomised phase III trial (NCT01591135) with a larger sample size is currently examining the efficacy of paclitaxel plus 5-FU compared with cisplatin plus 5-FU with concurrent radiotherapy employing a unified dose of 61.2 Gy at our hospital. In that study, the patient inclusion criterion was local advanced oesophageal squamous cell carcinoma (T2N0M0-TxNxM1a, AJCC 2002). Any patients with exploratory thoractomy, radiotherapy, chemotherapy or targeting therapy were excluded.

## Conclusions

In conclusion, our study indicates that weekly infused paclitaxel and continuously infused 5-FU for 72 h, followed by 2-cycle consolidation chemotherapy, is a promising regimen with good tolerability for patients with oesophageal cancer. Our study is limited by the small sample size. A current analysis of the efficacy of paclitaxel plus 5-FU compared to cisplatin plus 5-FU is ongoing at our hospital.

## Abbreviations

3DCRT: 3-dimensional conformal radiation therapy
5-FU: 5-fluorouracil
CT: Computed tomography
CTV: Clinical target volume
ECOG: Eastern cooperative oncology group
GTV: Gross tumour volume
IMRT: Intensity-modulated radiation therapy
NCI-CTC: National cancer institute common toxicity criteria
OS: Overall survival
PF: Cisplatin with 5-fluorouracil
PFS: Progression-free survival
PTV: Planning target volume
TP: Paclitaxel and cisplatin
